# ‘*Candidatus* Phytoplasma solani’ Predicted Effector SAP11-like Alters Morphology of Transformed Arabidopsis Plants and Interacts with AtTCP2 and AtTCP4 Plant Transcription Factors

**DOI:** 10.3390/pathogens13100893

**Published:** 2024-10-11

**Authors:** Marina Drcelic, Andreja Skiljaica, Bruno Polak, Natasa Bauer, Martina Seruga Music

**Affiliations:** Department of Biology, Faculty of Science, University of Zagreb, Horvatovac 102A, HR-10000 Zagreb, Croatia; marina.drcelic@biol.pmf.hr (M.D.); andreja.skiljaica@gmail.com (A.S.); polakbruno@yahoo.com (B.P.); natasa.bauer@biol.pmf.hr (N.B.)

**Keywords:** ‘*Candidatus* Phytoplasma solani’, BIFC, interaction, plant pathogens, SAP11 effector, TCP transcription factors, transgenic arabidopsis

## Abstract

Phytoplasmas are obligate intracellular pathogens that profoundly modify the development, physiology and behavior of their hosts by secreting effector proteins that disturb signal pathways and interactions both in plant and insect hosts. The characterization of effectors and their host-cell targets was performed for only a few phytoplasma species where it was shown that the SAP11 effector alters plant morphology by destabilizing plant transcription factors: TEOSINTE BRANCHED 1-CYCLOIDEA-PROLIFERATING CELL FACTOR (TCPs). To explore the possible role of the SAP11-like effector from ‘*Ca.* P. solani’, we used *Arabidopsis thaliana* as a model plant. The *SAP11-like* effector gene from ‘*Ca.* P. solani’ was introduced into arabidopsis by floral dip and transgenic lines were regenerated. *In planta* bimolecular fluorescence complementation (BIFC) assays in agroinfiltrated *Nicotiana benthamiana* leaf cells were conducted to detect interactions between SAP11-like and AtTCP2 and AtTCP4 using confocal microscopy. SAP11-like from ‘*Ca.* P. solani’ induced significant phenotypic changes in arabidopsis, including crinkled leaves with reduced size, lower biomass, more axillary branches, changes in root morphology, and crinkled and smaller siliques. The BIFC assays proved *in planta* interaction of SAP11-like effector with AtTCP2 and AtTCP4. To our knowledge, this is the first characterization of the interaction between the ‘*Ca.* P. solani’ effector and plant transcription factors, suggesting a potential mechanism of modulating plant development and induction of characteristic symptoms in ‘*Ca.* P. solani’-infected plants.

## 1. Introduction

‘*Candidatus* Phytoplasma’ (class Mollicutes), commonly known as phytoplasmas, are pleomorphic intracellular pathogens responsible for numerous economically significant plant diseases worldwide. For example, the losses associated with phytoplasma infections reported in the Czech Republic were 60% for tomato, 93% for pepper, and 100% for celeriac [1], while in only one year, an apple proliferation disease outbreak in Italy caused losses of up to EUR 100 million [2]. In nature, phytoplasmas are transmitted through insect vectors that feed on plant sap, and the complex life cycles of these bacteria invariably involve parasitizing both plant hosts and insects [3,4]. The ability of phytoplasmas to infect and multiply within a wide range of host cells suggests that these bacteria have developed mechanisms to influence cellular processes in both types of eukaryotic hosts [5]. In plant hosts, infection often leads to a range of symptoms that cause significant developmental changes, such as alterations in pigment production (yellowing), shifts in leaf morphogenesis [6] and abnormalities in flower formation (virescence). This can include the transformation of flower organs into leafy structures (phyllody), which results in plant sterility, excessive growth of side shoots (witches’ broom), and various other phenotypic changes. These symptoms typically culminate in the drying out and eventual collapse of the plant [7]. In vitro cultivation of these bacteria still remains unsuccessful, so studies on the mechanisms and strategies of pathogenicity and characterization of protein effector virulence rely mainly on functional genomics approaches [8,9,10].

In general, pathogen effectors are small molecules that selectively bind to host proteins or, in some cases, mRNA, thereby regulating their biological activity, such as enzyme activity, gene expression, or cellular signal transduction [3,11,12,13,14]. Unlike most bacterial pathogens, phytoplasmas secrete effectors directly into the host cell cytoplasm via the Sec-dependent system. The role of interactions and their mechanisms has been studied and characterized for only a few phytoplasma effectors so far [15]. Therefore, it is known that some can degrade MADs-box transcriptional factors (MTFs), such as SAP54 [16,17], or destabilize TEOSINTE BRANCHED 1-CYCLOIDEA-PROLIFERATING CELL FACTOR (TCP) transcriptional factors, such as SAP11 and its homologs [9,12,13,18,19].

‘*Ca.* P. solani’ (ribosomal group 16SrXII-A) is an endemic phytoplasma of the Euro-Mediterranean basin [20]. It is mostly associated with grapevine disease, *Bois noir*, and solanaceous diseases, known as stolbur disease [21] and maize redness [22]. In our previous studies, 38 potential effector genes were identified by sequencing and a detailed comparative genome analysis of the SA-1 strain of ‘*Ca.* P. solani‘ [23]. Among these, we have identified the SAP11-like effector, a homolog of the already described SAP11, one of the 56 secreted AY-WB proteins (SAPs) that were found in the Aster Yellows Witches’ Broom phytoplasma strain (AY-WB). Interestingly, virulence protein effector SAP11 showed the ability to destabilize a certain group of plant transcription factors (TCPs) [14,24,25]. The results of such interaction are visible in phenotype changes of the model plant *A. thaliana,* such as witches’ brooms and leaf shape changes [8,25,26,27]. Similar interactions of the ‘*Ca.* P. mali’ SAP11-like protein and TCPs have been characterized [19,28,29].

Therefore, within this study, we investigated the following question: whether the plant host phenotype changes when the ‘*Ca.* P. solani’ SAP11-like effector interacts with plant host proteins. Finally, the aim of this research was to reveal the possible role of the SAP11-like effector protein of ‘*Ca.* P. solani’, as, to the best of our knowledge, no data exists on any ‘*Ca.* P. solani’ effector interactions with the host cell targets and the associated molecular mechanisms.

## 2. Materials and Methods

### 2.1. Plant Growth

Seeds of wild-type *Arabidopsis thaliana* (L.) Heynh. ecotype Col-0 and *Nicotiana benthamiana* were surface sterilized with 70% ethanol for 1 min, and then subsequently with 1% Izosan G (100% sodium dichloroisocyanurate dihydrate, Pliva, Zagreb, Croatia) and 0.1% Mucasol™ (Sigma-Aldrich, St. Louis, MO, USA) for 10 min, rinsed five times with sterile distilled water, and plated on a Murashige and Skoog (MS) growth medium [30]. Plates with seeds were cold stratified for 72 h at 4 °C and incubated in 16 h light/8 h dark cycles (150 μmol/m^2^s light intensity) at 24 °C. After 15 days, seedlings were transplanted into the soil (Steckmedium KLASMANN, Klasmann-Deilmann GmbH, Geeste, Germany) and kept in 16 h light/8 h dark cycles (70 μmol/m^2^s light intensity) or 8 h light/16 h dark cycles (70 μmol/m^2^s light intensity) and 50% relative humidity at 21 °C [28]. The combination of two fluorescent tubes, PHILIPS TL-D Super80 830 (warm white) and 865 (cool white), was used for lighting in the 16 h light/8 h dark cycle experiments, while for the 8 h light/16 h dark cycle experiments, a plant growth chamber RK-500 CH (Kambič d.o.o., Semič, Slovenia) with OSRAM L-36W/TT Fluora fluorescent tubes was used.

### 2.2. Codon Optimization, Cloning, and Transformation of Arabidopsis Plants

The signal peptide sequence of SAP11-like (previously published SAP11-like, Acc. No. MPBG01000000, locus PSSA1_v1c1150) was predicted by using Signal IP v.3.0 [31]. The predicted cleavage site was between nucleotide positions 34 and 35. The coding sequence of SAP11-like without the signal peptide sequence was codon-optimized for expression in *A. thaliana* plants by using the GenSmart™ Codon Optimization tool (https://www.genscript.com/gensmart-free-gene-codon-optimization.html, accessed on 2 December 2022), inserted into pGEX-4T-1 vector by service GeneScript (Biotech, Princeton, NJ, USA), and used as template for PCR in subsequent cloning reactions. For the overexpression of *SAP11-like* in *A. thaliana* plants, the gene was amplified by PCR by using primers pGWB529-SAP11 fw/rev, purified by using NucleoSpin Gel and PCR Clean-up (MACHEREY-NAGEL Inc., Allentown, PA, USA), and subsequently cloned by using InFusion cloning technology (Clontech, Takara, San Jose, CA, USA) into the XbaI and SalI linearized binary plasmid vector pGWB529. All newly designed primers used for cloning are listed in Table S1. The primer pGWB529-SAP11 rev contained additional 18 nucleotides encoding a 6xHis-tag at the C-terminal end of the recombinant protein.

The binary plasmid was electroporated into *Agrobacterium tumefaciens* strain GV3101 (pMP90) and used for plant transformation via the floral dip method [32]. Transgenic plants were placed on an MS medium containing 30 mg/L of hygromycin B. Selected lines were selfed, and T3 transgenic progeny was used for experiments. To verify the presence of T-DNA insertion with the *SAP11-like* gene, DNA was isolated from 10-day-old seedlings from transformed and wild-type *A. thaliana* plants by using the OmniPrep™ for Plant (G-Bioscience^®^, St. Louis, MO, USA) Kit according to the manufacturer’s instructions. Approximately 80 mg of plant tissue per sample was used for extraction. Amplification of *SAP11-like* gene was performed by using specific primers pGWB529-SAP11 fw/rev (Table S1) with EmeraldAmp^®^ MAX PCR Master Mix (Takara Bio, Inc., San Jose, CA, USA) under the following PCR conditions: the initial denaturation step was set at 98 °C for 3 min, followed by 40 cycles of denaturation at 98 °C for 30 s, annealing at 58 °C for 30 s, extension at 72 °C for 1 min, and a final extension step at 72 °C for 7 min. All of the obtained amplicons were analyzed by using gel electrophoresis and sent for sequencing (Macrogen Europe BV, Amsterdam, The Netherlands) in order to be verified.

### 2.3. Phenotypic Analysis of Transgenic Arabidopsis thaliana Plants with SAP11-like Overexpression

The growth parameters of two independent transgenic lines overexpressing the *SAP11-like* gene were analyzed (2b and 3b) and compared to wild-type *A. thaliana*. We measured fresh shoot mass (mg), height (cm), rosette diameter (cm), the length and width (cm) of the three largest rosette leaves, the total number of axillary shoots, and the length of siliques in 45-day-old plants (*n* = 30 per line). We observed the phenotype and measured the length (cm) of 30 siliques in total, for each of the T3 lines and the wild-type. For each line and the wild-type, 5 siliques were sampled from 6 randomly chosen plants, approximately 5 cm from the inflorescence tip. Additionally, the root architecture was observed in plant seedlings 15 days after stratification.

Statistical analysis was conducted by using Student’s *t*-test, with differences between wild-type and transformed arabidopsis plants considered statistically significant if *p* < 0.05 and *p* < 0.0001.

### 2.4. Quantification of SAP11-like Gene Expression in Transgenic Arabidopsis thaliana Lines

RNA was isolated from 10-day-old seedlings (30 mg in total, 3b line, T3 generation) by using the RNeasy^®^ Plant Mini Kit (Qiagen, Hilden, Germany), following the manufacturer’s protocol. RNA was purified using the GenElute^™^ Total RNA Purification Kit (Sigma-Aldrich, Darmstadt, Germany) followed by DNAse I treatment, according to the manufacturer’s instructions. The concentration and purity of RNA samples were measured by using NanoDrop™ 2000/2000c Spectrophotometer (Thermo Fischer Scientific™ Waltham, MA, USA). For reverse transcription (RT), 500 ng of RNA in a final volume of 20 μL and 200 U of Maxima H Minus Reverse Transcriptase, 20 U RiboLock™ RNase Inhibitor, 5× RT Buffer, Oligo(dt)18 Primer, and dNTP Mix (Thermo Scientific™, Waltham, MA, USA) were used.

To distinguish complementary DNA (cDNA) from potential residual genomic DNA (gDNA), ACT3_fw/rev primers, together with the EmeraldAmp^®^ MAX PCR Master Mix (Takara Bio, Inc., San Jose, CA, USA), were used in a standard PCR reaction. The use of the ACT3 primer set enabled the amplification of the *ACT3* gene to discern cDNA (638 bp amplicon) from gDNA (732 bp amplicon) (Table S1).

For quantitative PCR (RT-qPCR), 1 µL of cDNA (10 ng/µL) was mixed with the GoTaq qPCR Master mix (Promega, Madison, WI, USA) and the designed primers 302Rv and 142Fw (Table S1). The house-keeping *A. thaliana* reference gene *ogio* (Acc. No. AT5G51880) [33] was used as an expression control and amplified by the primer pair ogioF (5′-ATCCAAGAGCAGTTCAAGCAAG-3′) and ogioR (5′-GAGAGCCATACCTTCCACTG-3′). Analysis was performed in duplicate on the MIC platform (Bio Molecular Systems, Upper Coomera, Australia) in a total reaction volume of 15 µL. The run profile of the PCR reaction was as follows: 95 °C for 5 min followed by 35 cycles of 95 °C for 5 s and 60 °C for 30 s. Melting curves were generated from 70 °C to 92 °C at a ramp speed of 0.1 °C/s to check for specific amplification.

### 2.5. Bimolecular Fluorescence Complementation (BiFC) in Nicotiana benthamiana Leaf Epidermal Cells

The *SAP11-like*, *AtTCP2,* and *AtTCP4* genes were amplified by PCR by using primers pSPYNE-SAP11 fw/rev (*SAP11-like*), pSPYCE-AtTCP2 fw/rev (*AtTCP2*), and pSPYCE-AtTCP4 fw/rev (*AtTCP4*), and purified by using NucleoSpin Gel and PCR Clean-up (MACHEREY-NAGEL Inc., Allentown, PA, USA). Purified fragments were subsequently cloned into BamHI linearized plasmids pSPYNE or pSPYCE [34] by using InFusion cloning technology (Clontech, Takara, San Jose, CA, USA) following the manufacturer’s instructions and with the primers listed in Table S1. For *SAP11-like* gene amplification, codon-optimized genes for *Nicotiana* sp. were synthesized and cloned into pGEX-4T (2.2) by the service GeneScript (Biotech, Princeton, NJ, USA) and used as the template, while AtTCP2 and AtTCP4 were amplified from previously constructed pDONR207 vectors [13].

Each plasmid (pSPYNE-SAP11-like, pSPYCE-AtTCP2, and pSPYCE-AtTCP4) was electroporated into the *A. tumefaciens* strain GV3101 (pMP90) together with *A. tumefaciens* containing the pCB301-p19 plasmid [35] and used for agroinfiltration of 6-8-week-old *N. benthamiana* plants [32] (Table 1). Agrobacteria transformed with pB7WGR2.0-EGFP-DMS3 were used for the determination of agroinfiltration efficacy [36]. Agrobacteria were grown overnight at 28 °C in 3 mL of LB liquid medium supplemented with respective selective antibiotics. They were subsequently pelleted at 4000 rpm for 20 min at room temperature and washed three times with 2.5 mL of 10 mM MES and 10 mM MgCl_2_ with pH = 5.3 buffer, followed by the addition of 150 μM of acetosyringone (final concentration). After 3–4 h of incubation at room temperature, agrobacterial suspensions were mixed (Table 1) and infiltrated into leaves [32].

After ~60 h, agroinfiltrated leaves of *N. benthamiana* were sampled for microscopic analyses by using the confocal laser scanning module TCS SP8 X FLIM (Leica Microsystems Wetzlar, Germany), fitted with a time-correlated single photon counting (TCSPC) unit (PicoQuant, Berlin, Germany) and an HC PL APO CS2 40×/OIL objective.

Samples of epidermal cells of the lower leaf surface from each plant were screened for YFP fluorescence (excitation 514 nm and emission at 516–549 nm) and chlorophyll autofluorescence (excitation 514 nm and emission at 654–735 nm). Detection of EGFP in the positive agroinfiltration control was obtained by using excitation at 488 nm and emission at a wavelength of 500–550 nm.

## 3. Results

### 3.1. Regeneration of Arabidopsis thaliana Plants Overexpressing SAP11-like Transgene

Approximately 1% of seeds germinated on a selective MS medium and developed into seedlings that were transferred into the soil. Very strong developmental changes were already observed in the first generation T1 of transgenic plants, where plants flowered, self-pollinated, and successfully produced seeds. Symptoms were visible in all independently transformed lines and through all (T1, T2, and T3) generations.

### 3.2. SAP11-like Gene Presence and Expression in Transformed A. thaliana Plants

The presence and identity of the *SAP11-like* gene were confirmed by successful amplification of the *SAP11-like* sequence of approximately 340 bp fragments from the DNA isolates of transformed arabidopsis plants, followed by sequencing (Figure S1).

**Figure 1:**
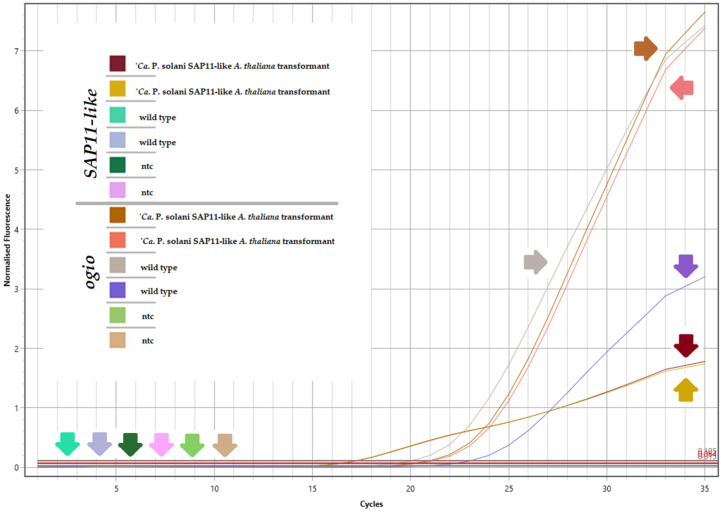
RT-qPCR: Amplification curves showing expression of *SAP11-like* gene sequence and reference gene sequence *ogio*. Samples of cDNA from ‘*Ca.* P. solani’ SAP11-like transformants and from wild-type arabidopsis. Colored arrows show amplification curves of corresponding samples.

To evaluate the expression levels of the *SAP11-like* gene, RT-qPCR was conducted. The housekeeping gene *ogio* was used for normalization. The low average Cq value of 16.32 for the *SAP11-like gene* and a large negative ΔCq of −3.85 compared to the *ogio* (Cq = 20.17) indicated a great expression level of the *SAP11-like* gene in transformed plants. As expected, no expression of the *SAP11-like* gene was detected in wild-type plants (Table 2, Figure 1).

### 3.3. SAP11-like Overexpressing Arabidopsis thaliana Plants Show Significant Phenotypic Changes

**Figure 2:**
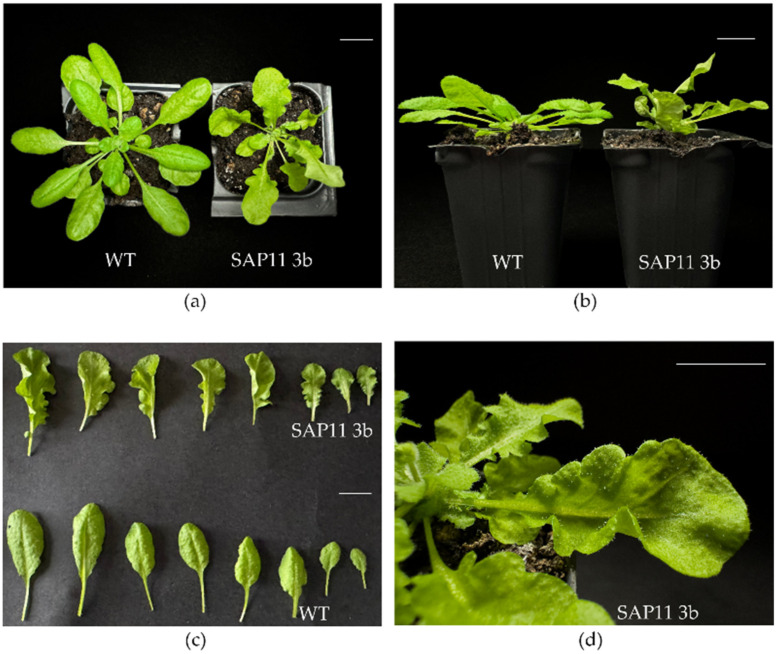
‘*Ca.* P. solani’s predicted effector SAP11-like alters the phenotype of transformed arabidopsis plants. (**a**) Wild-type, transformed 3b line, T3 generation arabidopsis. Transformed plants showing crinkled leaves and reduced growth (**a**,**b**). (**b**) Side view: wild-type (**left**) and transformed 3b line (**right**). (**c**) Comparison of arabidopsis rosette leaf morphology (**top**: transformed, **bottom**: wild-type). (**d**) Close-up of the transformed arabidopsis rosette showing crinkled leaves. Plants were grown in an 8 h light/16 h dark cycle. Bars: 1 cm.

**Figure 3:**
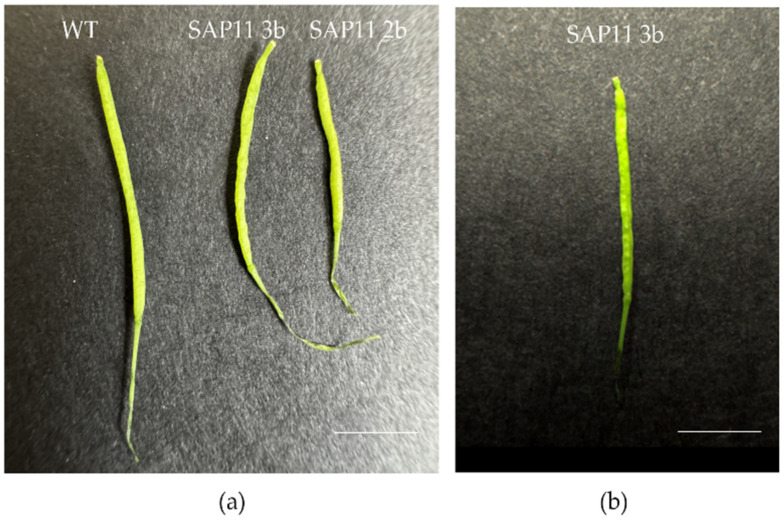
*A. thaliana* siliques phenotype differences 45 days after stratification. (**a**) Shorter siliques of SAP11-transformant lines compared to the wild-type. (**b**) Representative crinkled silique of SAP11-like transformant (3b line, T3 generation). Plants were grown in a 16 h light/8 h dark cycle. Bars = 0.25 cm.

**Figure 4:**
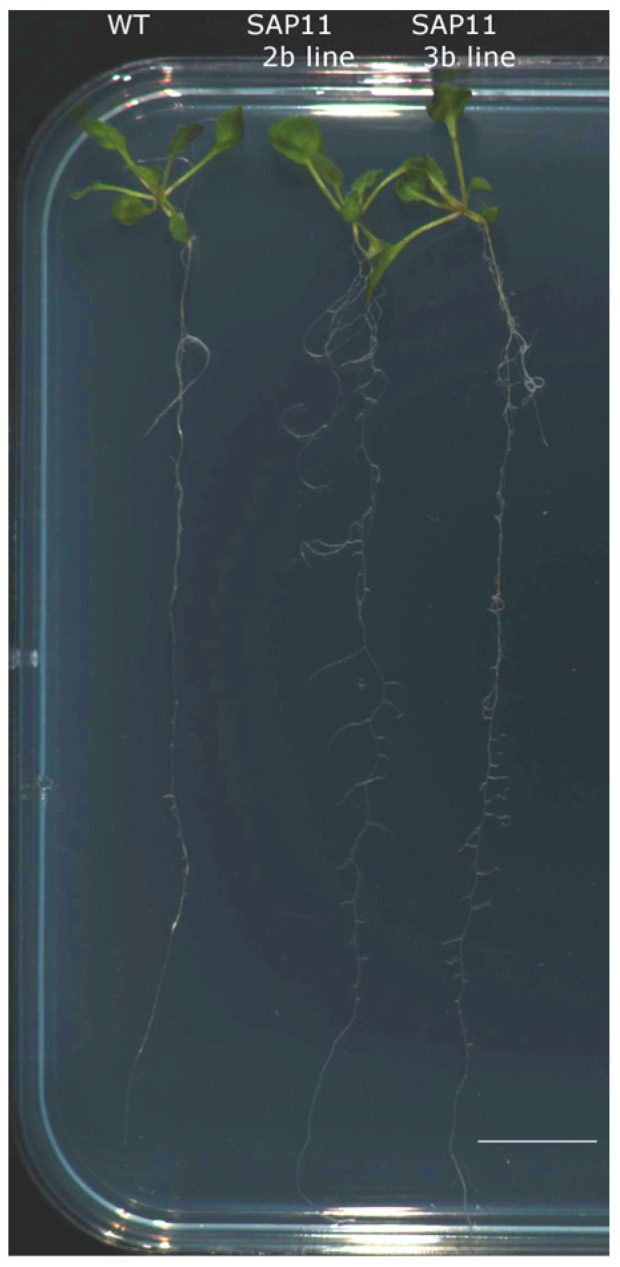
Comparison of *A. thaliana* root architecture in 15-day-old seedlings. Wild-type and SAP11-like transformant lines 2b and 3b. Seedlings were grown in a 16h light/8h dark cycle. Bar = 1 cm.

*A. thaliana* plants that were successfully transformed to overexpress the *SAP11-like* gene were compared to wild-type Col-0 plants. The SAP11-like transformants exhibited an unusual phenotype with altered leaf morphology of leaves being extremely crinkled and rolled, with reduced growth, crinkled and shorter siliques, irregular stem branching, and altered root morphology (Figure 2, Figure 3 and Figure 4).

Such changes were obvious from the early stages of plant growth (germination) (Figure S2) and were more expressed through the mid-stages (rosette growth; Figure 2) until the late stages of plant development (flowering). Forty-five days after stratification, all transformed arabidopsis plants had crinkled and rolled leaves without any exception (Figure 2). Compared to the wild-type, both lines 2b and 3b of SAP11-like transformants showed lower values of fresh shoot mass, height, rosette diameter, and the length and width of the three largest rosette leaves (Table 3, Figure S3). The total number of axillary shoots was higher than in the wild-type and showed a high significant difference (*p* < 0.0001). The SAP11-like 2b line had more pronounced phenotype changes (significant difference at *p* < 0.0001) for all measured parameters. The plants of the SAP11-like 3b line did not show significant differences in rosette diameter and width of rosette leaves but differed in average fresh shoot mass, height, length of rosette leaves and siliques, and total number of axillary shoots. (Table 3, Figure S3).

The average silique length of both (2b and 3b) SAP11-like transformants showed significantly lower values in comparison to the average wild-type silique length (Table 3), and although transgenic plants produced viable seeds, the seed number was reduced. Aside from being shorter, all siliques were also crinkled and deformed (Figure 3 and Figure S3).

In addition, significant root system architecture alternations were observed in 15-day-old transgenic seedlings. The increased number and intensive proliferation of lateral roots was noticed in transgenic seedlings of both lines compared to wild-type seedlings (Figure 4).

### 3.4. In Planta SAP11-like Interact with AtTCP2 and AtTCP4 Proteins

**Figure 5:**
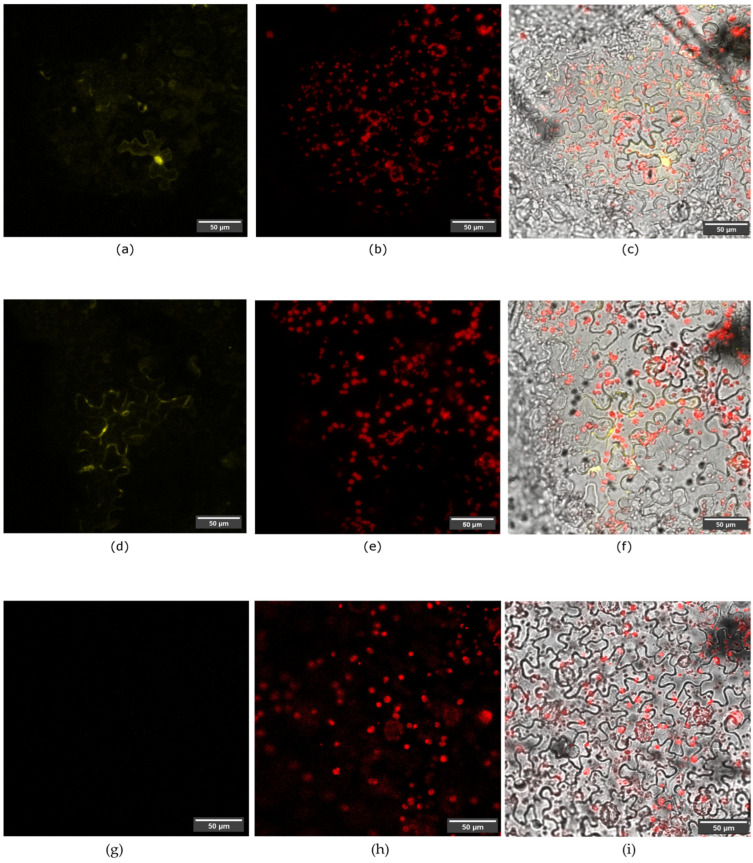
Detection of SAP11-like interaction with AtTCP2 and AtTCP4 in leaf epidermal cells of *N. benthamiana* using a BIFC assay. (**a**) YFP signal of SAP11-like interaction with AtTCP2 localized in cell cytoplasm and nuclei. (**b**) *N. benthamiana* chlorophyll. (**c**) Merged a and b with bright fields. (**d**) YFP signal of SAP11-like interaction with AtTCP4 localized in cell cytoplasm. (**e**) *N. benthamiana* chlorophyll. (**f**) Merged d and e with bright field. (**g**) Negative control (plasmids pSPYNE-SAP11-like and pCB301-p19) showing no YFP signal (Table 1). (**h**) *N. benthamiana* chlorophyll. (**i**) Merged g and h with bright fields.

The BIFC assay was conducted in agroinfiltrated *N. benthamiana* leaves, and the positive fluorescence signals (YFP) in both experimental combinations (Table 1) were detected. In the experiments of a potential interaction between the SAP11-like protein and AtTCP2, clear YFP signals were observed in both the nuclei and cytoplasm of agroinfiltrated leaf epidermal cells (Figure 5). On the other hand, in the experiments of the potential interaction between the SAP11-like effector and AtTCP4, clear YFP signals were detected only in the cell cytoplasm (Figure 5). No signal was detected in any of the negative controls (Figure 5), while in positive control, the detection of an EGFP signal demonstrated successful agroinfiltration (Figure S4). The detection of YFP signals in the BIFC assay experiments clearly demonstrated the interactions of the SAP11-like effector and the plant transcriptional factors AtTCP2 and AtTCP4 with different localization in agroinfiltrated epidermal cells.

## 4. Discussion

Upon the discovery of SAP11 homologs in genomes of many phytoplasma species, their properties, mechanisms, and functions have been thoroughly investigated, and it was even proposed that this effector could be considered “a universal phytoplasma effector” [19,37,38]. Regarding the mechanism and the interactions of SAP11 and SAP11-like proteins, it was shown that the small virulence effector SAP11 (approximately 10 kDa) from AY-WB phytoplasma binds to and destabilizes arabidopsis TCP transcription factors [8,24,39], which leads to significant alterations in leaf morphogenesis [25,40,41].

In our experimental system, the expression of the ‘*Ca.* P. solani’ SAP11-like protein (approximately 11 kDa) was under the control of the Cauliflower mosaic virus 35S promoter in transformed *A. thaliana*. It revealed similarities in symptom occurrence (Figure 2) with previously described characteristic phenotype changes in transformed *A. thaliana* expressing *SAP11* of ‘*Ca.* P. asteris’ [13,18,24] and ‘*Ca.* P. mali’ *SAP11* homolog [19,29,39].

The most dramatic phenotypic change we observed in the ‘*Ca.* P. solani’ SAP11-like transformed arabidopsis was in the leaf shape, where the leaves were profoundly crinkled (Figure 2), similar to previously described studies for SAP11 homologs from other phytoplasma species [24,27,29,42]. Their size, as well as the rosette diameter, was smaller compared to the wild-type plants. We also noticed that the fresh body mass was significantly lower in transformed arabidopsis than in wild-type plants (Table 3, Figure S3), even though the total number of axillary shoots was higher in both lines of transformed arabidopsis plants (Table 3, Figure S3). The average silique length was also lower in transformed plants than the average length of wild-type siliques (Table 3, Figure 3 and Figure S3), which had never been observed before, but it was noticed that seed germination ability had not been affected. Moreover, siliques were crinkled in the ‘*Ca.* P. solani’ SAP11-like transformed arabidopsis as previously noticed for the effect of SAP11 from AY-WB and the SAP11-like of ‘*Ca.* P. mali’ [24,28,39].

Similar to the witches’-broom symptom seen in phytoplasma-infected plants, arabidopsis plants transformed with the ‘*Ca.* P. solani’s *SAP11-like* gene showed enhanced proliferation of axillary shoots as well as stunted growth (Table 3), which was not reported before as an effect of the SAP11 effector of AY-WB [24]. Interestingly, only one of the SAP11-like homologs, SWP1 from Wheat Blue Dwarf phytoplasma, induced similar symptoms in transformed arabidopsis plants and interacted with AtTCP18 [42].

Moreover, in the transgenic SAP11-like arabidopsis seedlings obtained in this study, we observed the unusual proliferation of additional and lateral roots (Figure 3). Such change in root morphology was previously described in arabidopsis expressing the AY-WB SAP11 protein, where it was a result of an accumulation of cellular phosphate (Pi) and an upregulation of Pi starvation-induced genes and microRNAs. Also, it was shown that AY-WB SAP11 suppresses salicylic acid-mediated defense mechanisms, thereby promoting the growth of a bacterial pathogen. [27,43]. With this insight, further research on the role of the ‘*Ca.* P. solani’s SAP11-like effector in plant immune responses is needed. From that point of view, we suggest that there is a valid potential that the ‘*Ca*. P. solani’ SAP11-like could interact with other TCPs or interfere with miRNAs.

We demonstrated that the ‘*Ca.* P. solani’ SAP11-like protein interacts with two TCPs, the AtTCP2 and AtTCP4 proteins, when expressed in epidermal cells of agroinfiltrated *N. benthamiana* (Figure 5). Interestingly, it is known that AY-WB SAP11 localizes in plant nuclei, while the ‘*Ca.* P. mali’ SAP11 localizes in both the nuclei and cytoplasm [24,38,39,43]. In this research, we found that the interaction of the ‘*Ca.* P. solani’ SAP11-like with AtTCP2 was localized in both the nuclei and cytoplasm (Figure 5). However, in the case of the interaction between the ‘*Ca.* P. solani’ SAP11-like and AtTCP4 localization was observed only in the cytoplasm (Figure 5), unlike the interactions observed for AY-WB [38]. Since SAP11-like transgenic lines phenocopy multiple *tcp* mutant(s) [44], plant-specific SAP11 interaction partners and disrupted metabolic pathways in our regenerated transgenic plants will be investigated in more detail in the future.

Moreover, our results on ‘*Ca.* P. solani’s SAP11-like protein effect on the morphology of host plants and its specific interactions with plant targets would be directly applicable in agriculture and contribute to the scientifically-based development of new strategies for integrated plant protection of this significant pathogen. Since the only effective and available measures at the moment include the application of insecticides in order to control phytoplasma insect vectors [45,46], one of the potential control strategies could include RNAi-based technologies that have been previously shown to be effective in plant protection [47,48].

## 5. Conclusions

To the best of our knowledge, this is the first study regarding the characterization of the ‘*Ca.* P. solani‘ effector protein properties. Within this study, we have shown that the ‘*Ca.* P. solani‘ SAP11-like protein effector induces dramatic phenotype changes in transformed *A. thaliana* plants. Furthermore, the results obtained within this study suggest the potential role of the ‘*Ca.* P. solani’ SAP11-like effector in modulating TCP-related pathways, contributing to the observed phenotypic changes in transformed *A. thaliana* plants, in a manner as previously shown for some other phytoplasma species. This research provides novel evidence of the ‘*Ca.* P. solani‘ effector molecular mechanism and its pathogenicity impact on plant host organism. Furthermore, our results provide important data for new possibilities in finding solutions for the integrated pest management strategies and control of this important plant pathogen.

## Figures and Tables

**Table 1:** Combinations of recombinant plasmids used for *N. benthamiana* leaf agroinfiltration.

No. of *A. tumefaciens* Combination	Plasmid Constructs in Agroinfiltration Mixture	Type of Sample
1	pSPYNE-SAP11-like pSPYCE-AtTCP2 pCB301-p19	Experimental sample
2	pSPYNE-SAP11-like pSPYCE-AtTCP4 pCB301-p19	Experimental sample
3	pSPYNE-SAP11-like pCB301-p19	Negative control
4	pSPYCE-AtTCP2 pCB301-p19	Negative control
5	pSPYCE-AtTCP4 pCB301-p19	Negative control
6	pB7WGR2.0-EGFP-DMS3 pCB301-p19	Positive control of agroinfiltration

**Table 2:** RT-qPCR: Expression levels of *SAP11-like* gene in SAP11-like transgenic and wild-type *A. thaliana*.

Plant	Gene	Cq	Cq	Average Cq	ΔCq
*SAP11-like* transgenic *A. thaliana*	*ogio*	20.08	20.25	20.17	−3.85
	*SAP11-like*	16.34	16.29	16.32	
	ntc	0	0	0	Not applicable
Wild-type *A. thaliana*	*ogio*	19.18	22.19	20.69	Not applicable
	*SAP11-like*	0	0	0	
	ntc	0	0	0	

**Table 3:** Phenotypic analysis of transgenic *A. thaliana* plants with *SAP11-like* overexpression. Values shown are means ± se; *n* = 30 plants per line. All measurements were obtained 45 days after stratification. Plants were grown in a 16 h light/8 h dark cycle. Differences between wild-type (WT) and transformed arabidopsis plants are statistically significant if indicated by one (*p* < 0.05) or two (*p* < 0.0001) asterisks, as determined by Student’s *t*-test.

Measurement	WT	SAP11-like 2b	SAP11-like 3b
Fresh shoot mass ^1^ (g)	0.66 ± 0.06	0.27 ± 0.02 **	0.50 ± 0.06 *
Height (cm)	36.00 ± 0.99	23.79 ± 0.74 **	28.78 ± 0.91 **
Rosette diameter (cm)	6.42 ± 0.36	4.48 ± 0.19 **	6.09 ± 0.32
Length of rosette ^2^ leaf (cm)	2.19 ± 0.11	1.32 ± 0.05 **	1.94 ± 0.07 *
Width of rosette leaf ^2^ (cm)	1.16 ± 0.045	0.75 ± 0.03 **	1.03 ± 0.08
Length of siliques (cm)	1.34 ± 0.47	0.88 ± 0.23 **	0.89 ± 0.27 **
Ʃ axillary shoots	4.2 ± 0.33	9.56 ± 0.59 **	10.76 ± 0.85 **

^1^ Fresh shoot mass includes all aboveground arabidopsis plant tissues (i.e., the rosette and inflorescence); ^2^ The length and width of the three largest rosette leaves were measured for each plant (*n* = 30).

## Data Availability

The sequence SAP11-like_SA-1_PSSA1_v1c1150, Acc. No. MPBG01000000 is publicly available in GeneBank, NCBI.

## Supplementary Materials

The following supporting information can be downloaded at: https://www.mdpi.com/article/10.3390/pathogens13100893/s1, Figure S1: PCR confirmation of *SAP11-like* gene insertion in transformed *A. thaliana* plants. All obtained amplicons are approximately 340 bp. M—GeneRuler 1 kb DNA Ladder (Thermo Fischer Scientific™ Waltham, MA, USA); Figure S2: Leaf morphology differences in *A. thaliana* plants, 10 days after stratification; Figure S3: Average measurement values in *A. thaliana* plants, 45 days post seeds stratification (*n* = 30 for each sample). Differences between wild-type (WT) and transformed arabidopsis plants are statistically significant if indicated by one (*p* < 0.05) or two (*p* < 0.0001) asterisks, as determined by Student’s *t*-test; Figure S4: Positive control for agroinfiltration of *N. benthamiana* plants in BIFC assay (plasmids pB7WGR2.0-EGFP-DMS3 and pCB301-p19). (a) EGFP signal in epidermal cells. (b) *N. benthamiana* chlorophyll. (c) Merged a and b with bright fields; Table S1: List of primer sequences of genes used for cloning, PCR, and RT-qPCR.
