# Supplementary material for: ‘Candidatus Phytoplasma solani’ Predicted Effector SAP11-like Alters Morphology of Transformed Arabidopsis Plants and Interacts with AtTCP2 and AtTCP4 Plant Transcription Factors
Source: Pathogens. 2024 Oct 11;13(10):893. doi: 10.3390/pathogens13100893 (PMC11510232; doi:10.3390/pathogens13100893)
Supplement: Supplementary file 1 [file pathogens-13-00893-s001.zip › Table S1.pdf]

Table S1. List of primer sequences of genes used for cloning, PCR and RT-qPCR.

Gene	Primer name	Primer sequence 5′ – 3′ [1]	Details
SAP11-like	pGWB529-SAP11 fw	CACGGGGGAC**TCTAGA**ATGAATGTTTTCCTCTTCATTGGT	Cloning and verification of gene insertion by PCR
	pGWB529-SAP11 rev	GATCGGGGAAATTC**GAGCTC**TCAgtgatgatgatgatgatgCTTGTGACAATTGTCTGGT	
	pSPYNE-SAP11 fw	CGCCACTAGT**GGATCC**ATGAATGTTTTCCTCTTCATTGGTC	
	pSPYNE-SAP11 rev	TACTATCGAT**GGATCC**CTTGTGACAATTATCAGGCTGC	
AtTCP2	pSPYCE-AtTCP2 fw	CGCCACTAGT**GGATCC**ATGATTGGAGATCTAATGAAGAATAAC	
	pSPYCE-AtTCP2 rev	TACTATCGAT**GGATCC**GTTCTTGCCTTTACCCTTATG	
AtTCP2	pSPYCE-AtTCP4 fw	CGCCACTAGT**GGATCC**ATGTCTGACGACCAATTCC	
	pSPYCE-AtTCP4 rev	TACTATCGAT**GGATCC**ATGGCGAGAAATAGAGGAAG	
Actin 3	ACT3 fw	CTGGCATCATACTTTCTACAATG	Verification of contamination of cDNA with gDNA by PCR
	ACT3 rev	CACCACTGAGCACAATGTTAC	
SAP11-like	142 fw	GAGCTTTACAACACCTTGGAG	RT-qPCR
	302 rev	TTGTGACAATTGTCTGGTTGC	

[1] Restrictions sites used for cloning are in bold.
