# Supplementary figures and images for: ‘Candidatus Phytoplasma solani’ Predicted Effector SAP11-like Alters Morphology of Transformed Arabidopsis Plants and Interacts with AtTCP2 and AtTCP4 Plant Transcription Factors
Source: Pathogens. 2024 Oct 11;13(10):893. doi: 10.3390/pathogens13100893 (PMC11510232; doi:10.3390/pathogens13100893)

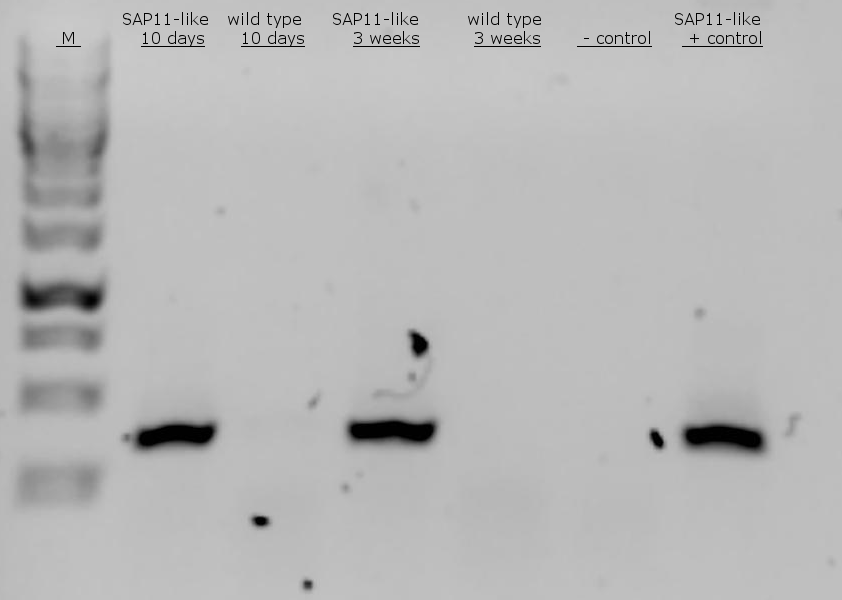

Supplement: Supplementary file 1 [file pathogens-13-00893-s001.zip › Figure S1.tiff]

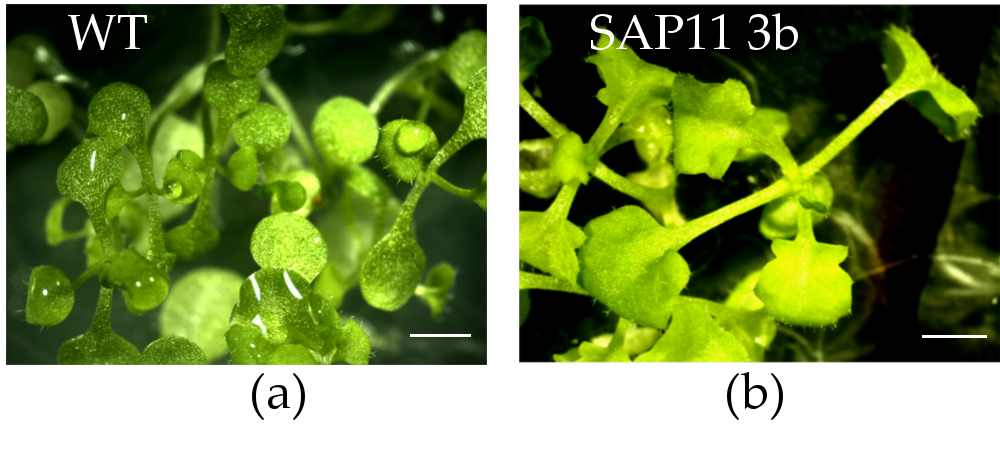

Supplement: Supplementary file 1 [file pathogens-13-00893-s001.zip › Figure S2.tiff]

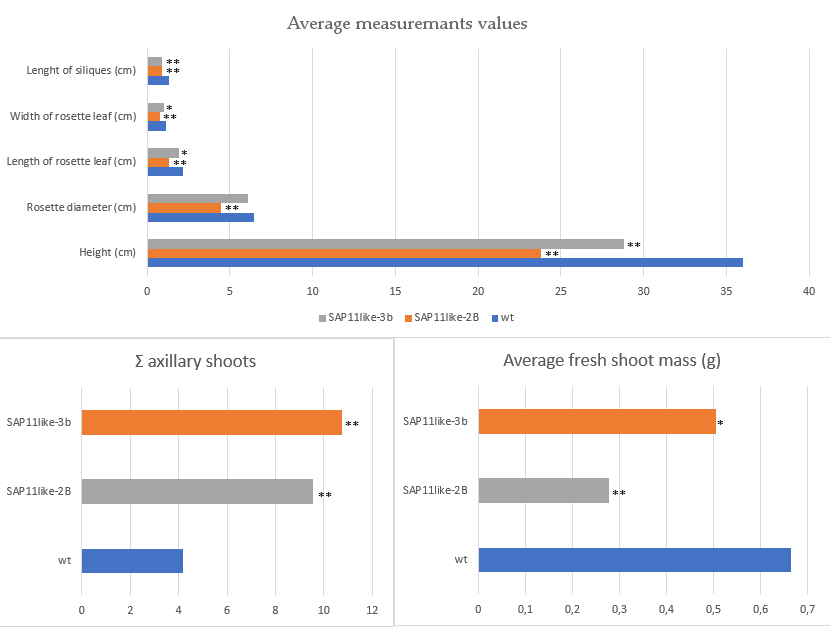

Supplement: Supplementary file 1 [file pathogens-13-00893-s001.zip › Figure S3.tiff]

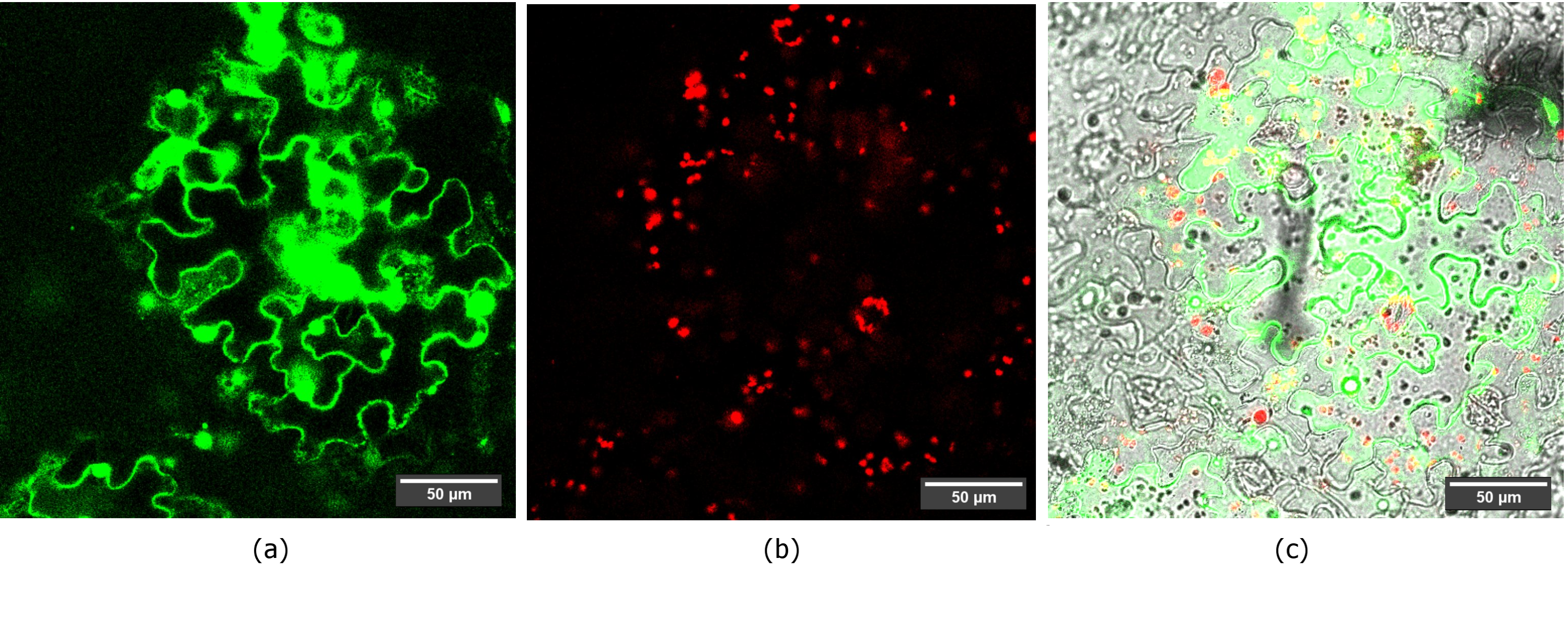

Supplement: Supplementary file 1 [file pathogens-13-00893-s001.zip › Figure S4.tif]
